# Frontal theta activation during motor synchronization in autism

**DOI:** 10.1038/s41598-017-14508-4

**Published:** 2017-11-08

**Authors:** Masahiro Kawasaki, Keiichi Kitajo, Kenjiro Fukao, Toshiya Murai, Yoko Yamaguchi, Yasuko Funabiki

**Affiliations:** 1 Department of Intelligent Interaction Technology, Graduate School of Systems and Information Engineering, University of Tsukuba, 1-1-1, Tennodai, Tsukuba-shi, Ibaraki 305-8573 Japan; 2 Rhythm-based Brain Information Processing Unit, RIKEN BSI-TOYOTA Collaboration Center, 2-1, Hirosawa, Wako-shi, Saitama, 351-0198 Japan; 3 Laboratory for Advanced Brain Signal Processing, RIKEN Brain Science Institute, 2-1, Hirosawa, Wako-shi, Saitama, 351-0198 Japan; 4 Department of Psychiatry, Graduate School of Medicine, Kyoto University, Yoshida-nihonmatsu-cho, Sakyo-ku, Kyoto, 606-8501 Japan; 5 Department of Psychology, Faculty of Human Sciences, Tezukayamagakuin University, 2-1823, Imakuma, Sayama-shi, Osaka, 589-8585 Japan; 6 Neuroinformatics Japan Center, RIKEN Brain Science Institute, 2-1, Hirosawa, Wako-shi, Saitama, 351-0198 Japan; 7 Department of Cognitive and Behavioral Sciences, Graduate School of Human and Environmental Studies, Kyoto University, Yoshida-nihonmatsu-cho, Sakyo-ku, Kyoto 606-8501 Japan

## Abstract

Autism is characterized by two primary characteristics: deficits in social interaction and repetitive behavioral patterns. Because interpersonal communication is extremely complicated, its underlying brain mechanisms remain unclear. Here we showed that both characteristics can be explained by a unifying underlying mechanism related to difficulties with irregularities. To address the issues, we measured electroencephalographm during a cooperative tapping task, which required participants to tap a key alternately and synchronously with constant rhythmic a PC program, a variable rhythmic PC program, or a human partner. We found that people with autism had great difficulty synchronizing tapping behavior with others, and exhibited greater than normal theta-wave (6 Hz) activity in the frontal cortex during the task, especially when their partner behaved somewhat irregularly (i.e. a variable rhythmic PC program or a human partner). Importantly, the higher theta-wave activity was related to the severity of autism, not the performance on the task. This indicates that people with autism need to use intense cognition when trying to adapt to irregular behavior and can easily become overtaxed. Difficulty adapting to irregular behavior in others is likely related to their own tendencies for repetitive and regular behaviors. Thus, while the two characteristics of autism have been comprehended separately, our unifying theory makes understanding the condition and developing therapeutic strategies more tractable.

## Introduction

Autism spectrum disorder (ASD) is characterized by two features in the Diagnostic and Statistical Manual of Mental Disorders (DSM-5) (American Psychiatric Association, 2013): persistent deficits in social interaction, and restricted, repetitive patterns of behavior, interests, or activities. Most ASD studies have focused on the first component, for aspects including imitation, language development, theory of mind, joint attention, and eye contact^1–8^. Some studies have shown cognitive inflexibility relevant to the second component, but these executive function studies did not include reciprocal interactive tasks^9–12^. To understand these two features of ASD in terms of a single underlying mechanism, we designed a rhythm-synchronization task featuring simple reciprocal communication with limited social demands. Previous studies have shown the behavioral synchronization in the nods during conversation^13^, the hand clapping^14^, and the tapping timing^15^, and so on. These synchronization plays important roles in facilitating and improving human relationship^16^.

Moreover, we included conditions that necessitated cognitive flexibility, because some studies have shown that the ASD people have inflexibility through executive functioning tasks^10–12^. Although we found no similar studies that investigated interpersonal behavioral synchronization in ASD during communication, recent studies have shown tight interpersonal synchronizations both in electroencephalograms (EEG) and behavioral rhythms in typical development (TD) individuals^17,18^.

Previous human neuroimaging studies have identified different mechanisms in the brain functions between ASD and TD, such as the emotion-related amygdala and the empathy-related mirror neuron systems (i.e., superior temporal sulcus)^19–22^. Moreover, the EEG studies showed the enhancements of theta synchronization (4-8 Hz) in ASD at both resting and cognitive states in comparison with that in TD participants^23,24^. However, no studies have examined how to modulate the brain activity with inter-person behavioral synchronization in ASD during communication.

Here, we found that synchronizing with the behavioral rhythms of others led to different behaviors and brain activities in ASD and TD individuals. We used the DSM-5^25^ for diagnosing ASD, and the Autism Diagnostic Observation Schedule (ADOS)^26^ and the Multi-dimensional Scale for Pervasive developmental disorder and Attention deficit/hyperactivity disorder (MSPA)^27^ for assessing its features and degree. We administered a task that required two participants (divided by a partition and unseen by each other) to alternate tapping a key back and forth. The participant pairs were either ASD-TD or TD-TD. During the task, we recorded electroencephalograms.

## Materials and Methods

### Participants

We recruited 24 ASD adults (14 men, 10 women; Age: 29.2 ± 7.2 years), Mean intelligence quotients were: full-scale IQ (FIQ) = 111 ± 11, Verbal IQ (VIQ) = 114 ± 12, and Performance IQ (PIQ) = 105 ± 13. ASD participants were without physical complications, psychosis or medication and were recruited from outpatient clinics or colleges in our city. TD controls were 24 adults (12 men and 12 women) who were age, sex, and IQ-matched to the ASD group (Age = 25.5 ± 6.6 years, FIQ = 111 ± 12, VIQ = 109 ± 14, PIQ = 110 ± 11). Other inclusion criteria were: FIQs ≥ 80 (for sufficient understanding of the study directions) and no medication (to avoid affecting the electroencephalogram (EEG). All participants gave written informed consent. Trained psychiatrists confirmed diagnosis of ASD according to the criteria in DSM-5. IQ was assessed with the Wechsler Adult Intelligent Scale-Third Edition^28^. We used data from the Wechsler Intelligence Scale for Children-Third Edition^29^ for a 20-year-old patient and the Wechsler Adult Intelligent Scale-Revised^30^ for another patient because the data were already available within the last 5 years. This study was approved by the RIKEN Ethical Committee and Kyoto University Ethical Committee in accordance with the Declaration of Helsinki. All participants gave written informed consent before participation in the study.

### ASD Assessment

We administered the following assessment batteries in order to grasp the degree and individual differences of autistic and comorbid features. Trained staff conducted the ADOS with supervision by specialists with research licenses, and the MSPA. The ADOS is a well-known assessment tool for autism. It consists of semi-structured tasks and interviews that involve communication and social interaction. We used module 4, which is for older adolescents and adults and takes 40–60 min to administer. The MSPA is a comprehensive assessment tool encompassing not only social communication of core ASD features but also other features such as restricted interests/behavior, sensory, inattention and motor problems that may relate to the present study. While the ADOS focuses on the behavior of social communication within the session, the MSPA includes the developmental history gathered from several informants and the objective behavior. The MSPA consists of 14 domains: five core domains (communication, social adaptation, empathy, restricted interests/behavior, stereotyped/repetitive motion), gross and fine motor, three attention-deficit/hyperactivity (ADHD) domains (inattention, hyperactivity and impulsivity), sensory, sleep cycle, learning, and language development. Each domain is assessed on a scale of one to five with half-point steps, according to the severity: one (no sign), two (somewhat but no need to support), three (clinical level: mild), four (moderate), five (severe).

We also used the autism-spectrum quotient (AQ) to assess the severity of ASD. Independent of the above assessments, participants filled out the form by themselves. Thus, we scored the severity of ASD in multiple ways of self-report, observation and interviews.

### Experimental procedure

The tapping task included three alternate tapping conditions: Human, Constant-PC, and Variable-PC. Throughout the task, participants wore headphones, closed their eyes, and sat in a chair alone in an electric- and sound-shielded room.

In the Human condition, two participants tapped their respective keys back and forth with their right index fingers. When a participant tapped, a sound (“do” or “mi”) was presented through both right and left headphones. When the partner tapped, the other sound (“mi” or “do”) was presented through both right and left headphones. If the difference between the previous tapping interval (e.g., from participant A to participant B) and the current one (e.g., from participant B to participant A) was within 50 ms, the one-octave higher sound was presented. Participants were instructed to tap the key at a time interval equal to that of the partner’s. The tapping rhythms were not predetermined or directed. Each participant was required to tap 250 times per session (total 500 taps/session).

In the Constant-PC condition, a participant performed the same task with a virtual person (a PC program) that they thought was the human partner. The PC generated taps at a constant interval (600 ms after the participants’ taps). In the Variable-PC condition, the tapping interval of the PC program varied from 400 to 800 ms in two patterns. For both patterns, the intervals changed after blocks of 50 trials. Pattern 1 was 600 ms → 400 ms → 600 ms → 800 ms → 600 ms and pattern 2 was 600 ms → 800 ms → 600 ms → 400 ms → 600 ms. Each person completed 250 trials for both of these conditions.

Each participant in the ASD and TD groups completed three or four separate sessions, respectively (ASD group: one session each for the Constant-PC, Variable-PC, and Human conditions; TD group: one session each for the Constant-PC, and Variable-PC conditions, and two sessions for the Human condition). In the Human condition, the partner for an ASD participant was always from the TD group, whereas the partner of a TD participant was either from the ASD or TD group. The results of ASD and TD participants were equally compared with each other under the same condition (with the same TD partner). The order of conditions was random.

For all conditions, participants were asked before the experiments to sit still without moving for about 2 s (baseline). All participants underwent a training session for the Human condition before the corresponding EEG measurement session. The stimulus was generated on a Windows computer using Matlab (Mathworks, Inc., Natick, MA, USA) with the Psychophysics Toolbox extension. Each sound was highly distinctive.

### EEG recordings and analyses

Individual EEG data were recorded continuously with 27 active scalp electrodes embedded in an electro cap (actiCAP) (Brain Products, Germany) in accordance with the international 10/10 system. EEG signals were re-referenced digitally according to the averaged recordings from the right and left earlobes. Electrode impedance was maintained below 14.6 kΩ. The vertical electrooculography (EOG) was recorded from electrodes that were placed above and below the left eye to monitor eye blinks or vertical eye movements. The horizontal EOG was recorded from electrodes that were placed 1 cm lateral from the right and left eyes. The EEG and EOG signals were amplified by BrainAmp ExG MR equipment (Brain Products, Germany). The sampling rate was 1000 Hz.

To reduce or eliminate artefacts, we conducted infomax independent components analysis (ICA) on the EEG data^31^. The ICA components with the most significant correlations with the vertical and horizontal EOGs were rejected, and the ICA-corrected data were recalculated by regression of the remaining components.

Next, to identify cortical activity with reduced effects of volume conduction, we applied a current source density transformation to the voltage distribution on the surface of the scalp using the spherical Laplace operator^32^.

Finally, to identify the time-frequency phases, wavelet transforms using Morlet’s wavelet function were applied^33^. We conducted the wavelet analyses for EEG data for each time epoch which was segmented into 3-sec epochs around the onset of button press (i.e. from −1.5 to 1.5 sec from button press). After that, we segmented the EEG data during the 0.5-sec epochs around the onset of button press (i.e. from −0.25 to 0.25 sec from button press), to minimize the edge artifacts of the low-frequency wavelets and to assure the epoch durations which are sufficient for the low-frequency analyses. The total number of epochs was 1000 epochs per one session for each participant (i.e. 500 taps × 2 participants). The number of epochs were almost same in all participants in both groups.

Morlet’s wavelets were used for the high time and frequency resolutions, which allowed a better observation of transitions in both low- and high-frequency oscillations. The amplitude for each time point during the tapping and observation periods was the arctangent of the result of the convolution of the original EEG signal *s*(*t*) with a complex Morlet’s wavelet function *w*(*t*, *f*):

$$w(t,f)=\sqrt{f}exp(-\frac{{t}^{2}}{2{\sigma }_{t}^{2}})exp(i2\pi ft)$$

where *σ*_*t*_ is the standard deviation of the Gaussian window (the number of cycles = 6), with *f* ranging from 1 to 20 Hz in 1-Hz steps. We used the same wavelets on different time and frequency points.

### Statistical analyses

The behavioral performance was evaluated by the Two-way analysis of variance (ANOVA) for groups (ASD vs. TD) and conditions (constant PC, variable PC, and Human). Furthermore, we analysed the Pearson’s correlations (N = 48 for total; N = 24 for each ASD and TD group; two-tailed) of the performance with 3 IQ scores and 18 ASD assessment scores (3 ADOS scores, 14 MSPA scores, and AQ score). We calculated the false discovery rate (FDR) corrected q-values^34^, instead of the uncorrected p-values. In the same way, we calculated the FDR q-values in the correlations of the EEG amplitudes with synchronization rate, ID scores and ASD scores.

## Results and Discussions

**Figure 1:**
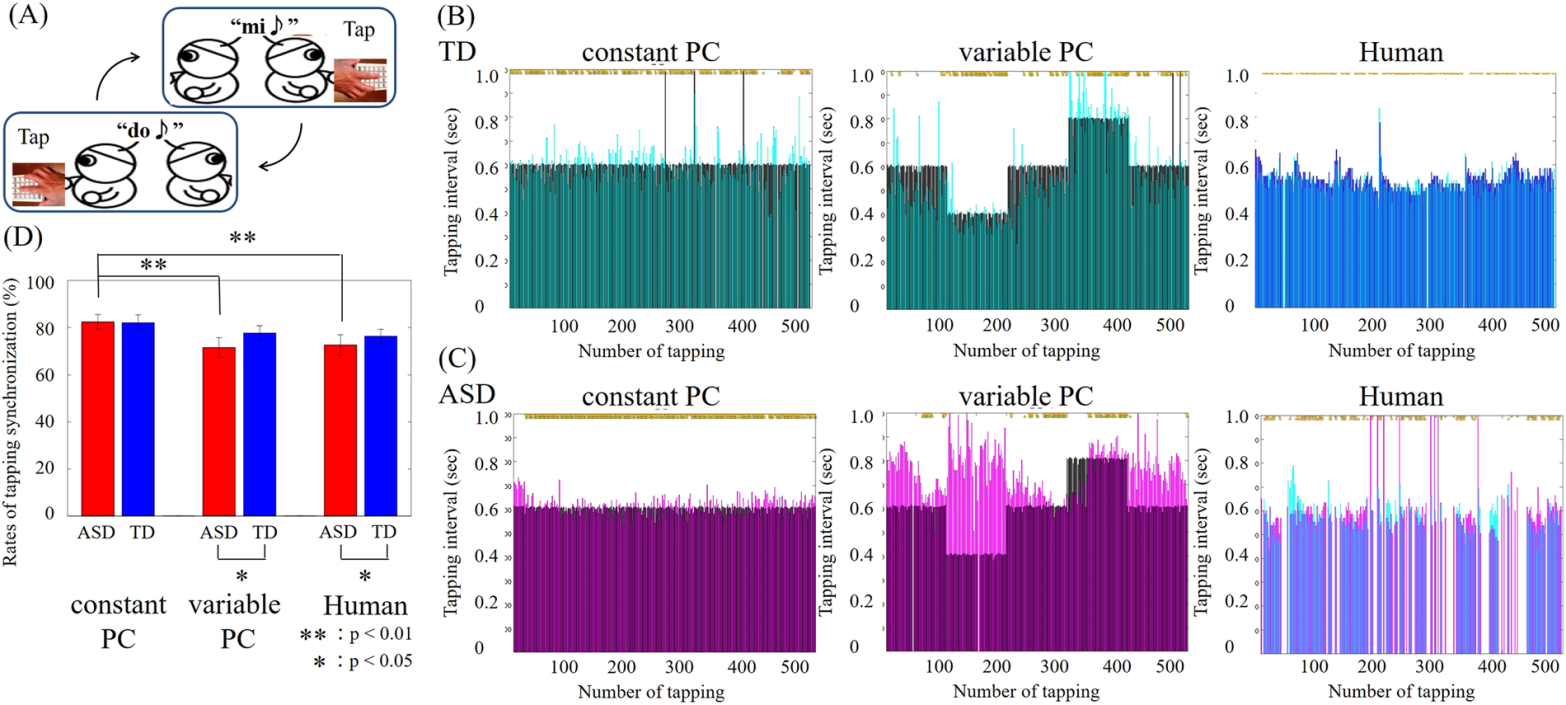
(**A**) Schematic illustration of the alternate tapping task. (**B**) Sample performance of a TD participant in the three conditions. *Cyan*, TD participant. *Black*, virtual partner. *Blue*, human partner. The vertical axis is the interval between one partner’s tap and the other’s. The horizontal axis represents the number of taps over time. (**C**) Sample performance of an ASD participant on the three conditions. *Purple*, ASD participant. *Blue*, same human participant as in (**B**). (**D**) Averaged rates of tapping synchronization for all participants within each group. Error bars denote the s.e.m. Significant differences between conditions were evaluated by Mann–Whitney U test.

Behavioral performance was evaluated by the rates of synchronized tapping. Examples of tapping intervals for the ASD and TD groups are shown in Fig. 1. Two-way analysis of variance (ANOVA) for groups (ASD vs. TD) and conditions (constant PC, variable PC, and Human) showed the main effects of conditions (*F*(2,138) = 3.07; *p* < 0.05) but no main effect of groups (*F*(1,138) = 1.19; *p* = 0.28) and interaction (*F*(2,138) = 0.42; *p* = 0.65).

The rates of synchronization in the ASD group were significantly lower than those in the TD group under the Human condition (mean ± sd: ASD: 72.50% ± 4.39%; TD: 76.18% ± 3.00%; Z = 1.85, *p* < 0.05, Mann–Whitney U test) and the Variable-PC condition (ASD: 71.38% ± 4.22%; TD: 77.61% ± 3.04%; Z = 2.20, *p* < 0.05, effect size = 0.45). In contrast, no significant differences between groups were observed in the Constant-PC condition (ASD: 82.26% ± 3.14%; TD: 81.94% ± 3.44%; Z = −0.28, *p* = 0.39, effect size = 0.06). Moreover, only in the ASD group did the performance significantly differ between the Constant-PC and Variable-PC conditions (Z = 2.96, *p* < 0.01, effect size = 0.60) and between the Constant-PC and Human conditions (Z = 3.18, *p* < 0.01, effect size = 0.65). These results show that individuals with ASD had greater difficulty in adapting to sudden fluctuations in partner behaviors, which are usual in daily communication.

To investigate which feature of ASD contributed to the synchronization rate, we calculated Pearson’s correlations between the rate and the severity of each ASD feature taken from the 3 main domains of the ADOS and all 14 domains of the MSPA. The FDR corrected q-values of correlations with IQ scores and ASD assessment scores were shown at Table 1A and B, respectively. Analysis showed that rates of synchronization were significantly correlated with ADOS and MSPA scores under Variable-PC and Human conditions (Table 1A). Synchronization rates in ASD participants correlated with MSPA scores for only restricted interests/behaviors in the Variable-PC and Human conditions (*q* < 0.05, sample size = 48). Additionally, synchronization rates correlated with MSPA scores for communication skill and empathy, and ADOS scores for reciprocal social interaction skill in the Variable-PC condition (all *q*_*s*_ < 0.01, sample size = 48). In human condition, synchronization rates correlated with MSPA scores for social adaptation (*q* < 0.01, sample size = 48). In contrast, no such correlations were found for ASD participants in the Constant-PC condition or in any condition for the TD group.

**Table 1:** The averaged IQ scores (A)/ASD assessment scores (B) of ASD and TD participants. The q-values (FDR-corrected p-values) from the correlation analyses between the IQ scores (A)/ASD assessment scores (B) and the rates of tapping synchronization for all participants (total), ASD, and TD groups under the constant PC (con.PC), variable PC (vari.PC) and human conditions (*: *q* < 0.05; number of comparison = 1 for Behavior, = 3 for IQ scores, = 18 for ASD assessment scores).

	ASD	TD	Total correlation			Total correlation			TD correlation		
	scores	scores	con.PC	vari.PC	Human	con.PC	vari.PC	Human	con.PC	vari.PC	Human
(**A**)											
IQ	111.33 ± 2.29	111.00 ± 2.49	0.544	0.984	1	1	0.931	0.986	1	0.893	0.529
verbal IQ	113.96 ± 2.35	109.75 ± 2.79	0.655	0.970	1	0.750	0.544	0.892	0.648	1	1
performance IQ	105.29 ± 2.50	110.04 ± 2.29	0.728	1	0.883	0.826	0.432	1	0.562	0.953	0.526
(**B**)											
communication	3.13 ± 0.16 *	1.40 ± 0.07	0.910	0.079	0.462	0.413	0.034*	0.170	1	1	0.959
social adaptation	3.23 ± 0.11 *	1.40 ± 0.08	0.897	0.068	0.549	0.724	0.061	0.038*	1	1	0.887
empathy	2.98 ± 0.12 *	1.42 ± 0.07	0.939	0.065	0.602	0.672	0.047*	0.145	1	0.988	0.847
restricted interests/behavior	3.38 ± 0.13 *	1.79 ± 0.09	0.921	0.089	0.361	0.556	0.034*	0.045*	1	0.964	0.993
Sensory	2.33 ± 0.16 *	1.42 ± 0.10	1	0.725	0.875	0.643	1	0.944	1	1	0.977
stereotyped/repetitive motion	1.58 ± 0.15 *	1.02 ± 0.02	0.850	0.682	0.938	1	0.973	0.885	0.993	1	0.659
gross motor	1.94 ± 0.16 *	1.25 ± 0.07	1	0.249	0.400	0.711	0.830	0.318	0.981	0.722	0.871
fine motor	1.65 ± 0.15	1.46 ± 0.09	0.803	0.517	0.676	0.812	0.839	0.420	1	0.934	0.839
Inattention	2.92 ± 0.19 *	2.23 ± 0.14	0.905	0.958	0.737	1	0.996	0.917	0.962	0.981	0.972
hyperactivity	1.85 ± 0.15	1.46 ± 0.09	0.861	0.449	0.946	0.939	0.961	0.920	1	0.692	0.872
Impulsivity	2.19 ± 0.16 *	1.50 ± 0.11	1	0.180	0.832	0.652	1	1	1	0.736	1
sleep cycle	2.23 ± 0.18 *	1.46 ± 0.15	1	1	0.896	0.984	0.475	0.930	0.491	0.717	0.904
learning	1.73 ± 0.17 *	1.17 ± 0.08	1	0.117	0.831	0.616	0.087	0.395	0.413	0.554	0.564
language development	1.41 ± 0.15 *	1.30 ± 0.11	0.929	1	0.929	0.936	0.987	0.991	1	1	0.916
ADOS (communication)	2.17 ± 0.30 *	0.63 ± 0.14	0.793	0.076	0.549	0.777	0.120	0.209	0.908	0.995	1
ADOS (reciprocal social interaction)	4.67 ± 0.70 *	0.92 ± 0.25	1	0.007*	0.272	0.492	0.028*	0.153	0.599	0.607	0.844
ADOS (imagination and creativity)	0.75 ± 0.17 *	0.13 ± 0.07	0.915	0.249	0.924	0.942	0.513	0.924	1	1	0.888
AQ	17.09 ± 0.85 **	9.62 ± 0.89	1	0.066	0.625	1	0.494	1	0.620	0.711	0.826

**Figure 2:**
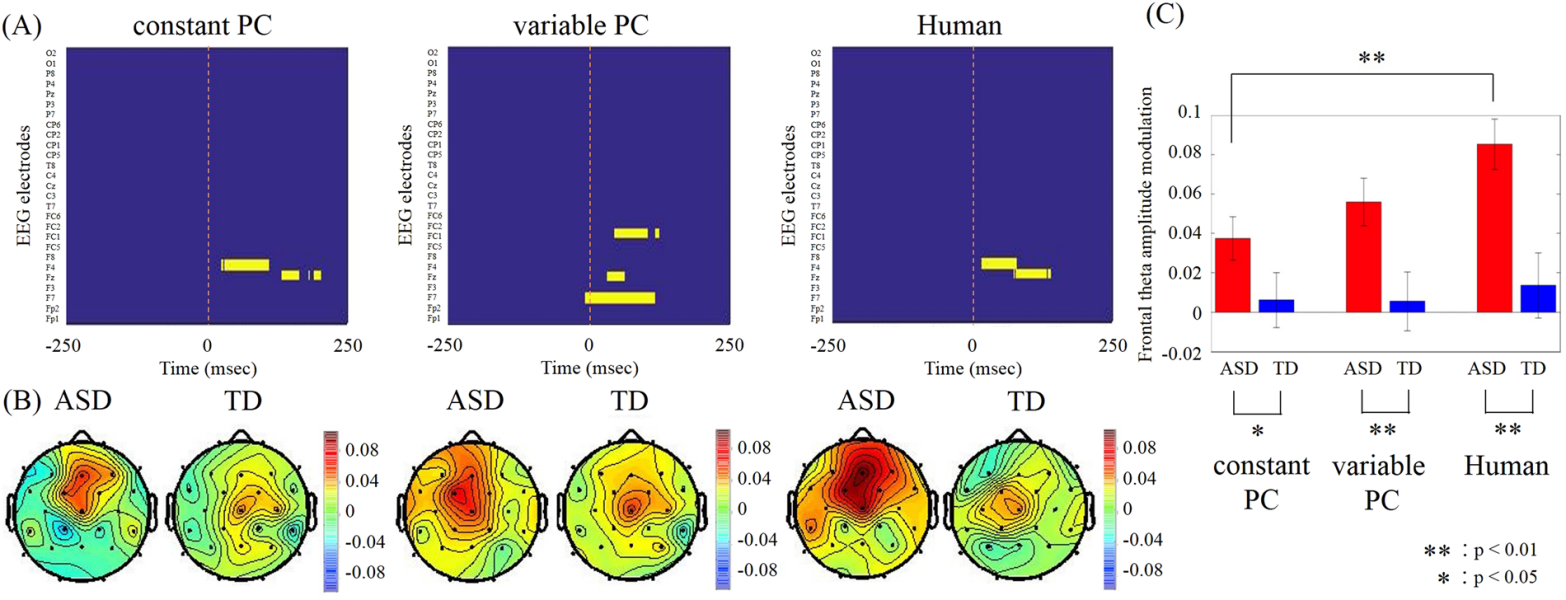
(**A**) Colour maps showing the channels and the time during the observation period in which significantly higher theta amplitudes were observed in the ASD group (yellow; *p* < 0.05, Mann-Whitney U-test with Bonferroni corrected for multiple comparisons). (**B**) Topographical maps for the averaged theta (6 Hz) amplitude modulations during the 0–50 ms after onset of the other’s tapping for TD and ASD groups. (**C**) Averaged frontal (Fz electrode) theta amplitude modulations during the same 0–50 ms. Error bars denote s.e.m. Significant differences between conditions were evaluated by Mann–Whitney U test.

**Figure 3:**
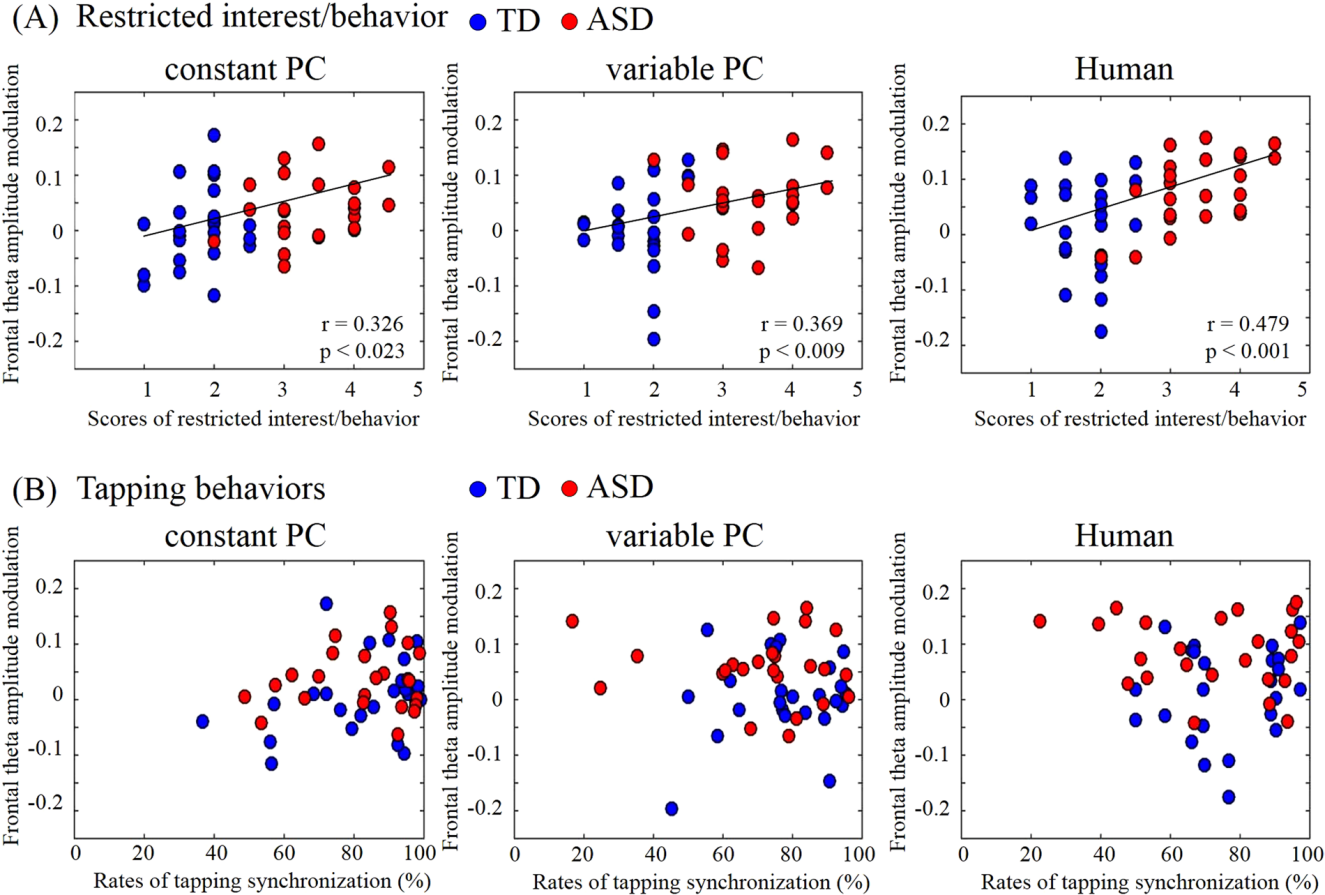
Scatter plots depicting the relationship of frontal theta-amplitude modulation with MSPA scores for restricted interest/behavior (**A**) and with the rates of tapping synchronization (**B**). The regression lines show high significance (Pearson correlation analysis; *p* < 0.01).

To examine the relationship between behavior and instantaneous brain activity, we conducted time-frequency analyses on the electroencephalogram data collected during tapping (tapping period) and while listening to partner tapping (observation period). We compared these data with those recorded during the inter-session interval (baseline period). In all three conditions, the ASD group showed increased theta activity in the midline prefrontal regions just after the onset of the observation period (from onset to 50 ms). Two-way ANOVA for groups and conditions showed the main effects of groups (*F*(1,138) = 20.20; *p* < 0.001) but no main effect of conditions (*F*(2,138) = 2.04; *p* = 0.13) and interaction (*F*(2,138) = 1.06; *p* = 0.35). No such increase was detected during the tapping period for either group, or during the observation period for the TD group. The theta activity in the ASD group was significantly higher than that during the same time window in the TD group (Fig. 2; different peak frequency: 6 Hz; *p* < 0.05, Bonferroni corrected for multiple comparisons). No other regions were significantly different between the groups. We calculated Pearson’s correlations between the frontal activity and ASD assessment scores. The FDR corrected q-values of correlations with synchronization rate, ID scores and ASD scores were shown at Table 2A,B, and C respectively. We found that the amount of theta activity positively correlated with MSPA communication skills, social adaptation, empathy, and restricted behaviors in the Human condition and with empathy and restricted behaviors in the constant PC condition (all *q*_*s*_ < 0.01). The sensory domain in the MSPA, which shows atypical sensory reactivity, did not relate the EEG activity. Figure 3 shows the relationship between theta activity and the restricted behavior with which it correlated the most. In contrast, no significant correlations were found between the frontal activity and synchronization rates.

**Table 2:** The q-values (FDR-corrected p-values) from the correlation analyses frontal theta (6 Hz) amplitude modulations (from the onset of partner tapping to 100 ms) and the rates of tapping synchronization (A), the IQ scores (B), or ASD assessment scores (C) for all participants (total), ASD, and TD groups under the constant PC (con.PC), variable PC (vari.PC) and human conditions (*: *q* < 0.05; number of comparison = 1 for Behavior, = 3 for IQ scores, = 18 for ASD assessment scores).

	Total correlation		
	con.PC	vari.PC	Human
(**A**)			
Behavior	0.121	0.850	0.295
(**B**)			
IQ	0.702	0.894	1
verbal IQ	0.690	1	4.390
performance IQ	1	4.502	0.849
(**C**)			
communication	0.203	0.149	0.020*
social adaptation	0.171	0.160	0.017*
empathy	0.042*	0.177	0.042*
restricted interests/behavior	0.043*	0.088	0.011*
Sensory	0.969	0.204	0.156
stereotyped/repetitive motion	0.834	0.146	0.106
gross motor	0.865	0.881	0.488
fine motor	0.863	0.559	0.322
Inattention	0.847	0.290	0.097
hyperactivity	0.897	0.481	0.215
Impulsivity	0.645	0.516	0.093
sleep cycle	0.893	0.518	0.319
learning	0.803	0.497	0.202
language development	0.799	0.546	0.195
ADOS (communication)	0.198	0.129	0.102
ADOS (reciprocal social interaction)	0.482	0.144	0.055
ADOS (imagination and creativity)	0.165	0.470	0.100
AQ	0.110	0.142	0.056

Decreased beta amplitudes and increased theta amplitude in the central electrodes which is closed to the motor area were commonly observed in all conditions and in both groups around the onset of the tapping period. These results were consistent with the finding that beta desynchronization is related to several motor movements, such as the voluntary hand movements^35^ and the synchronous hand movements^36^. Thus, the simple sensory-motor systems in ASD appear similar to those in TD for behaviors related to synchronization, which do not require empathy-like processes.

Notably, we also found large individual differences in the synchronization rate under the Variable-PC and Human conditions. The variance in the ASD group was particularly large; the best and the worst performers in the Human condition and the best two and the worst three performers (n = 48) in the Variable-PC condition were all from the ASD group. Indeed, not all ASD participants performed poorly on our synchronization task. This is unsurprising because ASD has a wide variety of features and comorbidities, and individual differences are large. However, interpretation of the variance is not complicated, because our task related only to specific cognitive functions (i.e., task switching, sensory-motor adaptation, cooperation with the behaviors of others). Our MSPA data showed that only communication, social adaptation, and restricted interests/behavior influenced the results, while other features such as fine motor coordination, stereotyped movement, sensory, attention, hyperactivity, impulsivity, had no effect on either behavioral performance or frontal theta activity.

Increased frontal theta activity is known as one of the brain areas which correlate with executive processing capacity^37–39^. Therefore, the theta elevation that we observed suggests that this communication might need cognitive resources on individuals with ASD. Interestingly, the increase in frontal theta activity was correlated with the severity of ASD, rather than with the behavioral performance, meaning that the frontal activation cannot reveal how much they struggled with the task. Instead, the results suggest that individuals with ASD must put effort into thinking and planning their communicative behaviors, while TD individuals synchronize their behaviors with others automatically.

Also, the frontal theta enhancements in the ASD group might reflect the difficulties in motor planning characteristic of ASD^40–43^. Although this study could not identify the exact brain regions from only the EEG recordings and analyses due to low spatial resolution, the theta-enhanced areas were close to the motor areas, especially the supplementary motor areas. The supplementary motor areas are thought to be associated with motor planning as well as motor execution^44^. Therefore, the ASD participants would be expected to have difficulty with motor planning as well as executive function.

The frontal theta activation was seen even when the tapping rhythm was regular. Because participants were not informed that the rhythm was regular, they might have consciously planned their tapping in response to the regular PC and were thus able to cope with it behaviorally. In the irregular conditions, simultaneously with further frontal activation, behavioral performance dropped. In addition to not being able to keep up with unexpected changes in rhythm made by the PC, ASD participants could not even cope with natural fluctuations made by a human partner or with changes made by the PC partner. Even natural human fluctuations have a irregular rhythm that might be difficult for ASD people to follow. Thus, communication difficulties of people with ASD might result from their inability to adapt to irregularity. ASD includes persistent deficits in social communication, as well as restricted and repetitive patterns of behavior, interests, or activities (define in DSM-5). Further, people with ASD are known to prefer objects to people to a greater extent than TD individuals do^45,46^. Assuming that human behaviors are essentially irregular, the first component of ASD (deficits in social communication) could be explained by this difficulty with irregularity. The second component (restricted and repetitive behavior) itself is a preference for the regular and can be viewed as intolerance of irregularity. Thus, we can see how this single underlying symptom might explain a fundamental mechanism of ASD.

Furthermore, because the frontal theta activity occurred just after the onset of the partner’s tapping, ASD participants might have been consciously confirming the other’s behavior and planning their own in response. The increased activity in the irregular conditions would suggest that individuals with ASD need more cognitive resources to make such strategies in unpatterned situations. In clinical practice, some ASD patients receive social skill training or behavioral interventions^47,48^. These therapies might boost cognitive compensation for difficulty in communication. In daily-life situations, individuals with ASD often need experience simulations to adapt to new or irregular environments. These findings increase our understanding of the mechanisms underlying ASD and the difficulties these patients have in daily life.
